# Insights from the Fungus *Fusarium oxysporum* Point to High Affinity Glucose Transporters as Targets for Enhancing Ethanol Production from Lignocellulose

**DOI:** 10.1371/journal.pone.0054701

**Published:** 2013-01-30

**Authors:** Shahin S. Ali, Brian Nugent, Ewen Mullins, Fiona M. Doohan

**Affiliations:** 1 Molecular Plant-Microbe Interactions Laboratory, School of Biology and Environmental Science, University College Dublin, Dublin, Ireland; 2 Department of Crop Science, Teagasc Crop Research Centre, Oak Park, Carlow, Ireland; Soonchunhyang University, Republic of Korea

## Abstract

Ethanol is the most-widely used biofuel in the world today. Lignocellulosic plant biomass derived from agricultural residue can be converted to ethanol via microbial bioprocessing. Fungi such as *Fusarium oxysporum* can simultaneously saccharify straw to sugars and ferment sugars to ethanol. But there are many bottlenecks that need to be overcome to increase the efficacy of microbial production of ethanol from straw, not least enhancement of the rate of fermentation of both hexose and pentose sugars. This research tested the hypothesis that the rate of sugar uptake by *F. oxysporum* would enhance the ethanol yields from lignocellulosic straw and that high affinity glucose transporters can enhance ethanol yields from this substrate. We characterized a novel hexose transporter (Hxt) from this fungus. The *F. oxysporum* Hxt represents a novel transporter with homology to yeast glucose signaling/transporter proteins Rgt2 and Snf3, but it lacks their C-terminal domain which is necessary for glucose signalling. Its expression level decreased with increasing glucose concentration in the medium and in a glucose uptake study the Km_(glucose)_ was 0.9 mM, which indicated that the protein is a high affinity glucose transporter. Post-translational gene silencing or over expression of the *Hxt* in *F. oxysporum* directly affected the glucose and xylose transport capacity and ethanol yielded by *F. oxysporum* from straw, glucose and xylose. Thus we conclude that this Hxt has the capacity to transport both C5 and C6 sugars and to enhance ethanol yields from lignocellulosic material. This study has confirmed that high affinity glucose transporters are ideal candidates for improving ethanol yields from lignocellulose because their activity and level of expression is high in low glucose concentrations, which is very common during the process of consolidated processing.

## Introduction

Industry continually seeks to improve the efficacy and processing costs associated with the production of ethanol from lignocellulosic plant material. While the current strategy is mainly based on pretreatment followed by enzymatic hydrolysis, fungal-mediated consolidated bioprocessing (CBP) of lignocellulosic material has significant potential to bring a breakthrough in commercial production by reducing the overall cost [1]. CBP involves all the four biologically-mediated transformations *viz.* the production of saccharolytic enzymes, the hydrolysis of carbohydrate components to simple sugars, the fermentation of hexose sugars and the fermentation of pentose sugars in a single step [2]. Fungi that have shown promise as CBP agents include *Fusarium oxysporum* [3], *Mucor hiemalis* [4], *Neurospora crassa* [5], *Rhizopus oryzae* [6] and *Trametes hirsute* [7]. *F. oxysporum* has been extensively studied in terms of its ability to produce ethanol from lignocellulosic biomass [3], [8], [9], [10], [11].

A previous study [8] showed that there is inter-strain variation within *F. oxysporum* in terms of the ability to produce ethanol from lignocellulosic material. However there was no obvious link between ethanol productivity and either cellulase or alcohol dehydrogenase activity [8]. One strain (11C) released 80% of the maximum theoretical ethanol yield from alkali-treated straw and 24% from untreated straw. At the other end of the spectrum, strain 7E released 54.4% and 4.4% ethanol from these two respective substrates [8]. Using the suppression subtractive hybridisation (SSH) technique, it was observed that there were many differences in gene activity between strains 7E and 11C during lignocelluose breakdown, some of which might account for differences in bioconversion efficacy [12].

Among the various genes up-regulated in strain 11C as compared to 7E, one encoded a glucose transporter (Hxt) and real-time RT-PCR analysis also showed that the expression level of this particular gene was several fold higher in strain 11C compared to 7E [12]. Transport across the cell membrane is the first step in the metabolism of sugars and this occurs through facilitated diffusion [13], [14]. It has been observed that the rate of sugar transport determines the rate of anaerobic fermentation in the yeast *Saccharomyces cerevisiae* [15]. Brandao & Loureiro-Dias [16] observed that sugar transport across the *F. oxysporum* membrane was under the same regulatory mechanism as that of yeast and other eukaryotic microorganisms. In *S. cerevisiae* there are 20 different glucose transporter genes and the expression of these transporters is largely regulated by the glucose concentration [17]. Each transporter plays a specific role because they all have different substrate specificities and affinities. Hxt1p and Hxt3p are the low-affinity carriers (Km_(glucose)_ 100 mM). Hxt6p and Hxt7p have a high affinity for their substrate (Km_(glucose)_ 1–2 mM), whereas Hxt2p and Hxt4p display only moderate to low affinity (Km_(glucose)_ ∼ 10 mM) [18]. The transporters differ not only in kinetic characteristics but also in their protein expression patterns [19]. Expression of the genes encoding these transporters is regulated not only by the available sugar concentration [17], [20], [21] but also by osmotic pressure [22], [23], starvation [24] and the physiological state of the cell [21], [25], [26], [27]. This complex regulation ensures that the yeast receives an adequate supply of carbon and energy under various conditions [28].

The objective of this study was to characterise the *Hxt* gene previously identified as being overexpressed in *F. oxysporum* during lignocellulose bioconversion to ethanol [12] and to determine if it affects ethanol productivity. The *F. oxysporum Hxt* gene was silenced and overexpressed in strain 11C. The resultant mutants were used in CBP studies in order to determine the effect of the encoded Hxt protein on the bioconversion of both a wheat straw/bran mix and simple sugars to ethanol. The effect of the *F. oxysporum Hxt* gene on yeast sugar transport was also determined.

## Results

### Cloning and Characterisation of the *F. oxysporum Hxt* Gene

**Figure 1:**
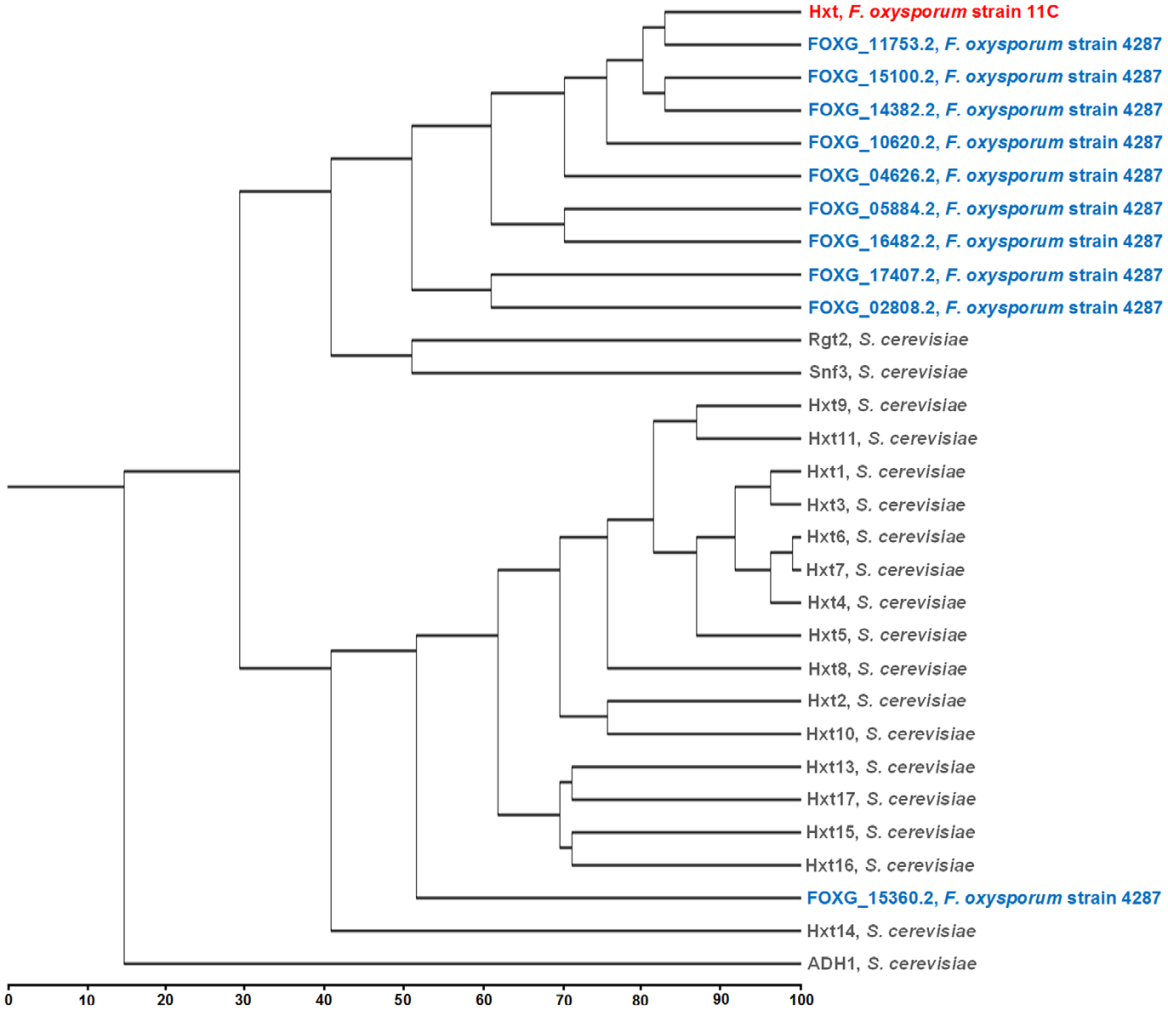
Dendrogram depicting the sequence similarity among the glucose transporter proteins from *Fusarium oxysporum* and the yeast *Saccharomyces cerevisiae.* The *F. oxysporum* strain 11C Hxt protein sequence was deduced from the nucleotide sequence and the yeast and *F. oxysporum* strain 4287 glucose transporter amino acid sequences were obtained from *Saccharomyces* Genome Database (www.yeastgenome.org) and *Fusarium* comparative genomics database (FCGD) (http://www.broadinstitute.org/annotation/genome/fusarium_graminearum) respectively. Protein sequences were aligned using European Bioinformatics Institutes’s ClustalW2 tool (www.ebi.ac.uk) [62] and the dendrogram was generated using the Unweighted Pair Group Method with Arithmetic mean (UPGMA) method [63].

The full-length *Hxt* mRNA was sequenced and the encoded ORF was determined to comprise 1596 nucleotides (GeneBank No. JX089403). The crystal structure of the deduced amino acid sequence highlighted 12 helices (using lactose permease (2cfqA) as a template) [29] and transmembrane (TM) domain analysis of the amino acid sequences also confirmed 12 TM helices with short C and N-terminal ends protruding into the cytoplasm (See Figure S1). The deduced amino acid sequences showed homology with ten different proteins encoded within the *F. oxysporum* f. sp. *lycopersici* (strain 4287) genome (http://www.broadinstitute.org/) (percent identity ≥30%) (Figure 1). Four of these are annotated as high affinity glucose transporters (FOXG_11753.2, FOXG_15100.2, FOXG_14382.2, FOXG_10620.2), four as monosaccharide transporters (FOXG_04626.2, FOXG_17407.2, FOXG_02808.2, FOXG_15360.2) and two as RCO3 (regulator of conidiation genes-3) (FOXG_05884.2, FOXG_16482.2) which are also involved in glucose transport [30]. The yeast *S. cerevisiae* is the organism in which glucose transporters are most fully characterised; it encodes 20 different transporters [17]. Of these, the yeast Rgt2 and Snf3 proteins showed highest homology (∼41%) to the *F. oxysporum* Hxt protein (Figure 1 and Figure S2). The *F. oxysporum* protein showed ∼30% homology to the other yeast Hxt proteins (Figure 1).

### Temporal Accumulation of *Hxt* Transcript during CBP of Wheat Straw/Bran

**Figure 2:**
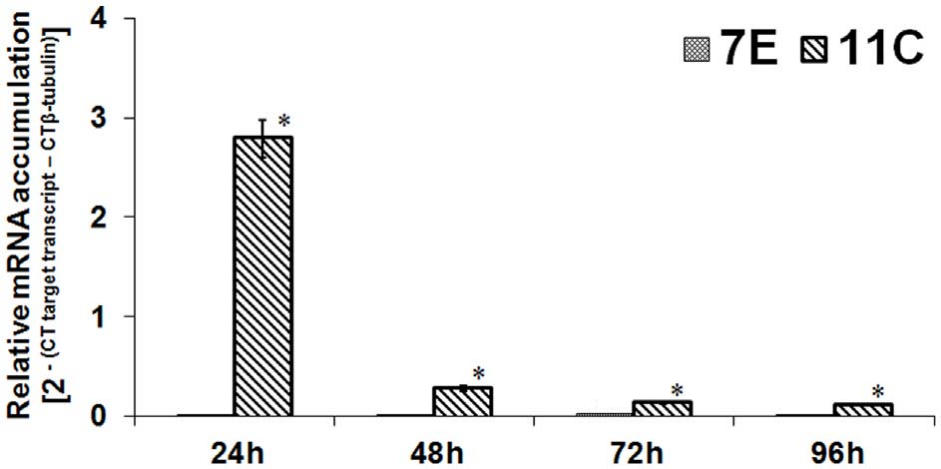
Temporal analysis of the accumulation of transcript encoding a high affinity glucose transporter (*Hxt*) during the saccharification of wheat straw/bran by *Fusarium oxysporum* strains 11C and 7E. *F. oxysporum* were aerobically cultured on wheat straw/bran (10∶1 ratio) and RT-PCR was conducted using RNA from samples harvested at either 24, 48, 72 or 96 h post-inoculation. *Hxt* transcript accumulation was quantified relative to that of the housekeeping gene β-tubulin (FOXG_06228.2). Results are based on two experiments, each with three replicates per treatment. Bars indicate Standard Error of Measurement (SEM). For any given time point, an ‘*’ above the columns indicates that values were significantly different between 7E and 11C at *P*≤0.05.

The *Hxt* transcript was originally identified as being up-regulated in *F. oxyporum* strain 11C as compared to 7E during the CBP of wheat straw/bran, 24 h post-fungal inoculation [12]. Real time RT-PCR was used to analyse the temporal accumulation of the *Hxt* transcript in these two strains of *F. oxysporum* during aerobic growth on wheat straw/bran, relative to that of the housekeeping gene *β*-tubulin (Figure 2). *Hxt* transcription was highly up-regulated in *F. oxysporum* strain 11C as compared to 7E, as determined by RT-PCR analysis (*P*≤0.05*)* (Figure 2). Transcript levels were highest at 24 h post-fungal inoculation and showed a sharp decline by 48 h (Figure 2).

### Effect of Sugars and Ethanol on *Hxt* Transcription

**Figure 3:**
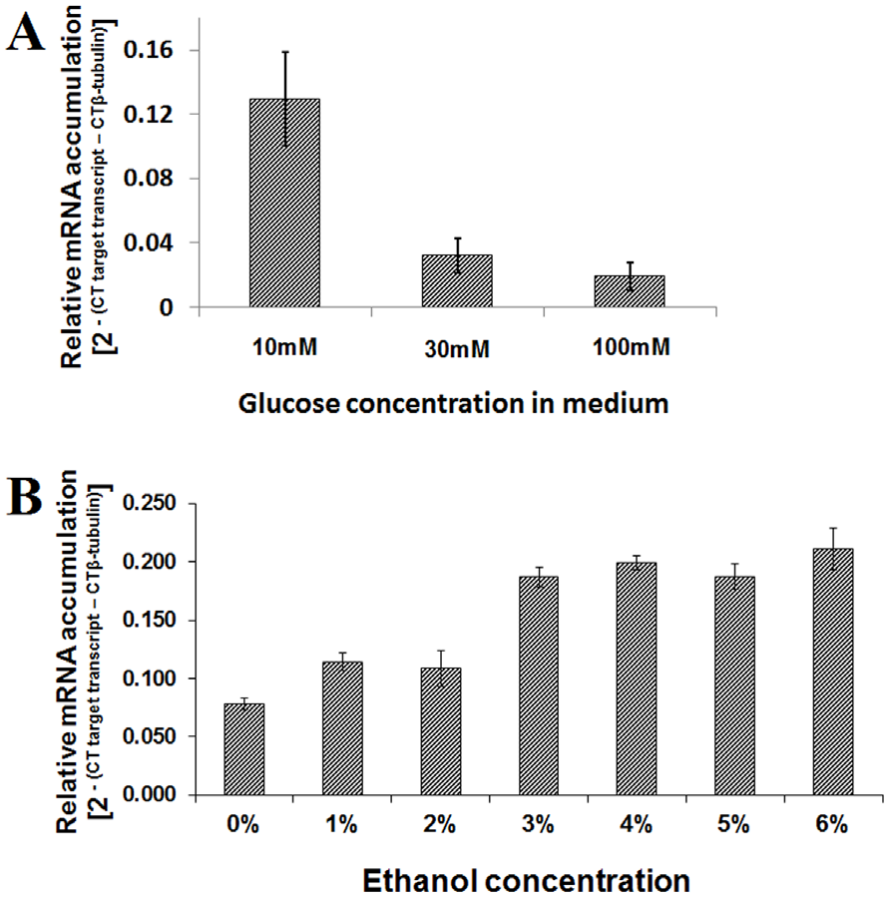
Effect of glucose and ethanol on the transcription of the *Fusarium oxysporum Hxt* gene encoding a putative hexose transporter. *F. oxysporum* strain 11C was grown in minimal media [51] supplemented with (**A**) 10–100 mM glucose as the sole carbon source, or (**B**) 20 mM glucose as the carbon source and 0–6% ethanol. Flasks were inoculated with fungal spores (10^5^ ml^−1^), plugged with sterile cotton wool and incubated at 30°C in a shaking incubator for 24 h at 150 rpm under dark and RNA was extracted from harvested mycelia. *Hxt* transcript accumulation was quantified relative to that of the housekeeping gene β-tubulin (FOXG_06228.2). Results are based on two experiments, each with three replicates per treatment. Bars indicate SEM (LSD_0.05_ A = 0.097; LSD_0.05_ B = 0.035).

Experiments were conducted in order to determine if glucose and ethanol regulate the transcription of the *F. oxysporum Hxt* gene, as they do for yeast *Hxt* genes [17], [28]. The *Hxt* mRNA levels were higher when the fungus was grown in 10 as compared to either 30 or 100 mM glucose as the sole carbon source (Figure 3A). *Hxt* transcript levels increased with increasing alcohol concentration, plateauing at 3% (Figure 3B).

### The Contribution of the *Hxt* Gene to Lignocellulose Bioconversion by *F. oxysporum*

**Figure 4:**
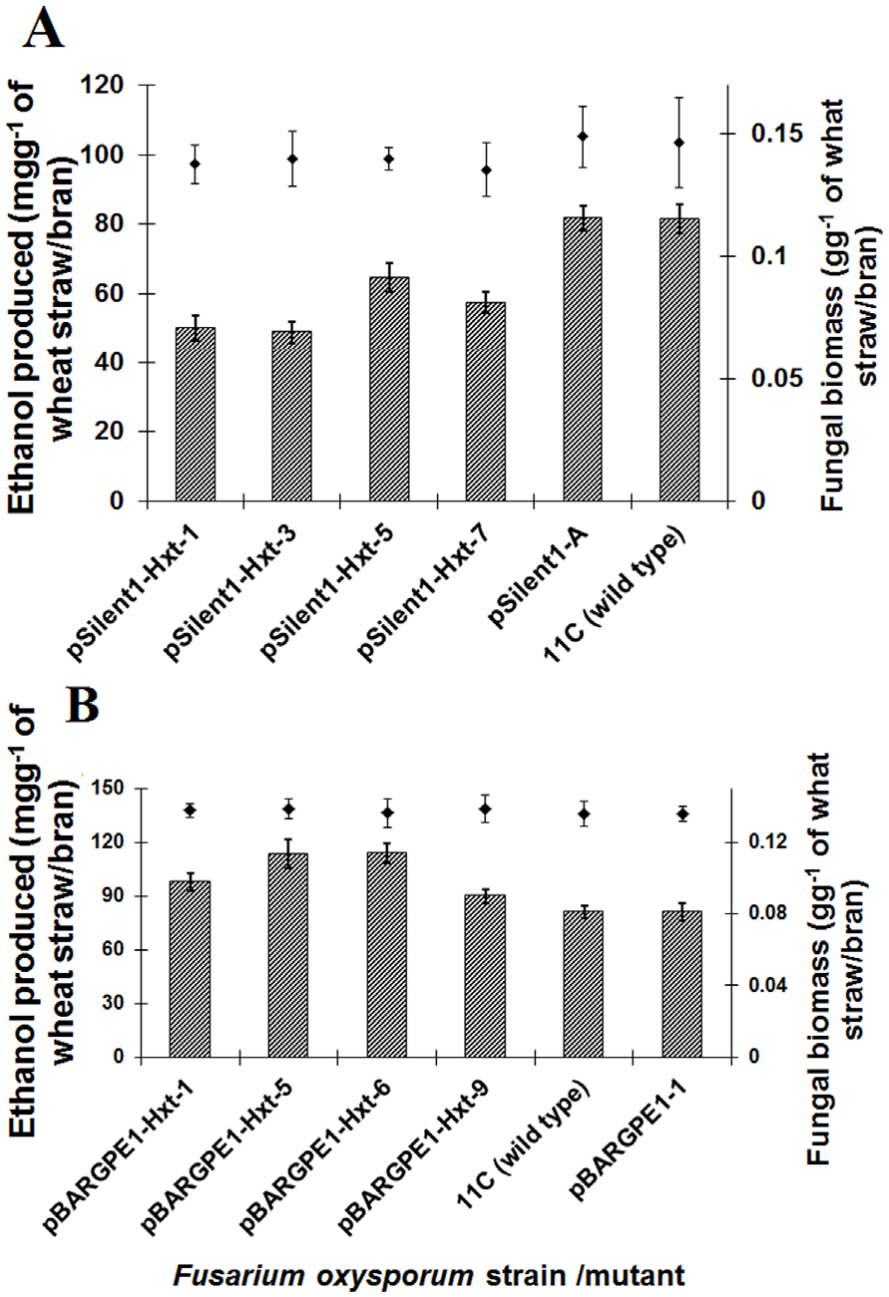
The effect of silencing and overexpressing the high affinity glucose transporter (*Hxt*) gene on the ability of *Fusarium oxysporum* strain 11C to colonise and release ethanol from a straw/bran mix (10∶1 ratio). (**A**) Gene silenced mutants pSilent-1-Hxt-1, 3, 5 & 7, mutant pSilent-1-*A* transformed with the empty silencing vector and wild type fungus; (**B**) gene overexpressed mutants pBARGPE1-Hxt-1, 5, 6 & 9, mutant pBARGPE1-1 transformed with the empty overexpression vector and wild type fungus. For both A and B, fungi were cultured on straw/bran for 96 h aerobic followed by 96 h oxygen-limiting growth. Ethanol was estimated using QuantiChrom™ Ethanol Assay Kit (DIET-500) (BioAssay Systems, USA). Results are based on two experiments, each with three replicates per strain/mutant. Bars indicate SEM [LSD_0.05_ A = 11.9 (ethanol), non-significant (biomass); LSD_0.05_ B = 15.16 (ethanol), non-significant (biomass)].

PTGS and gene overexpression via fungal transformation was used to respectively repress and up-regulate the function of the Hxt in *F. oxysporum* strain 11C (see Results S1). Southern hybridisation confirmed that all the silencing and overexpression mutants except mutant pBARGPE1-Hxt-5 contained a single copy of the vector integrated into the genomic DNA (see Figures S3 and S4) and real time RT-PCR analysis of transcript levels confirmed the efficacy of gene silencing and over expression (see Fig. S5). PTGS significantly reduced the amount of ethanol yielded by *F. oxysporum* via CBP of untreated straw/bran mix (*P*≤0.05) (Figure 4A). The four *Hxt-*silenced mutants tested yielded between 15 and 40% less ethanol compared to the wild type strain 11C or a mutant strain transformed with the empty vector (*P*≤0.05) (Figure 4A). There was a correlation between the level of transcript accumulation and ethanol production (*r = *0.913; n = 6; *P*≤0.05), but the silencing of this gene did not affect the amount of fungal biomass produced (*P*≥0.05) (Figure 4A).

Overexpression of *Hxt* gene significantly increased the ethanol yield by the fungus following CBP of a straw/bran mix (*P*≤0.05) (Figure 4B). There was almost a 39% increase in ethanol yield by the two mutants, pBARGPE1-Hxt-5 and pBARGPE1-Hxt-6, compared to the wild type strain 11C or a mutant strain transformed with the empty vector (Figure 4B). Like the silencing mutants, there was also a correlation between the level of transcript accumulation and ethanol production (*r = *0.968; n = 6; *P*≤0.050). However, overexpression of this particular gene did not affect the fungal biomass produced (*P*≥0.05) (Figure 4B).

### Effect of *Hxt* on Ethanol Yield from Sugars and Alkali-treated Straw

**Figure 5:**
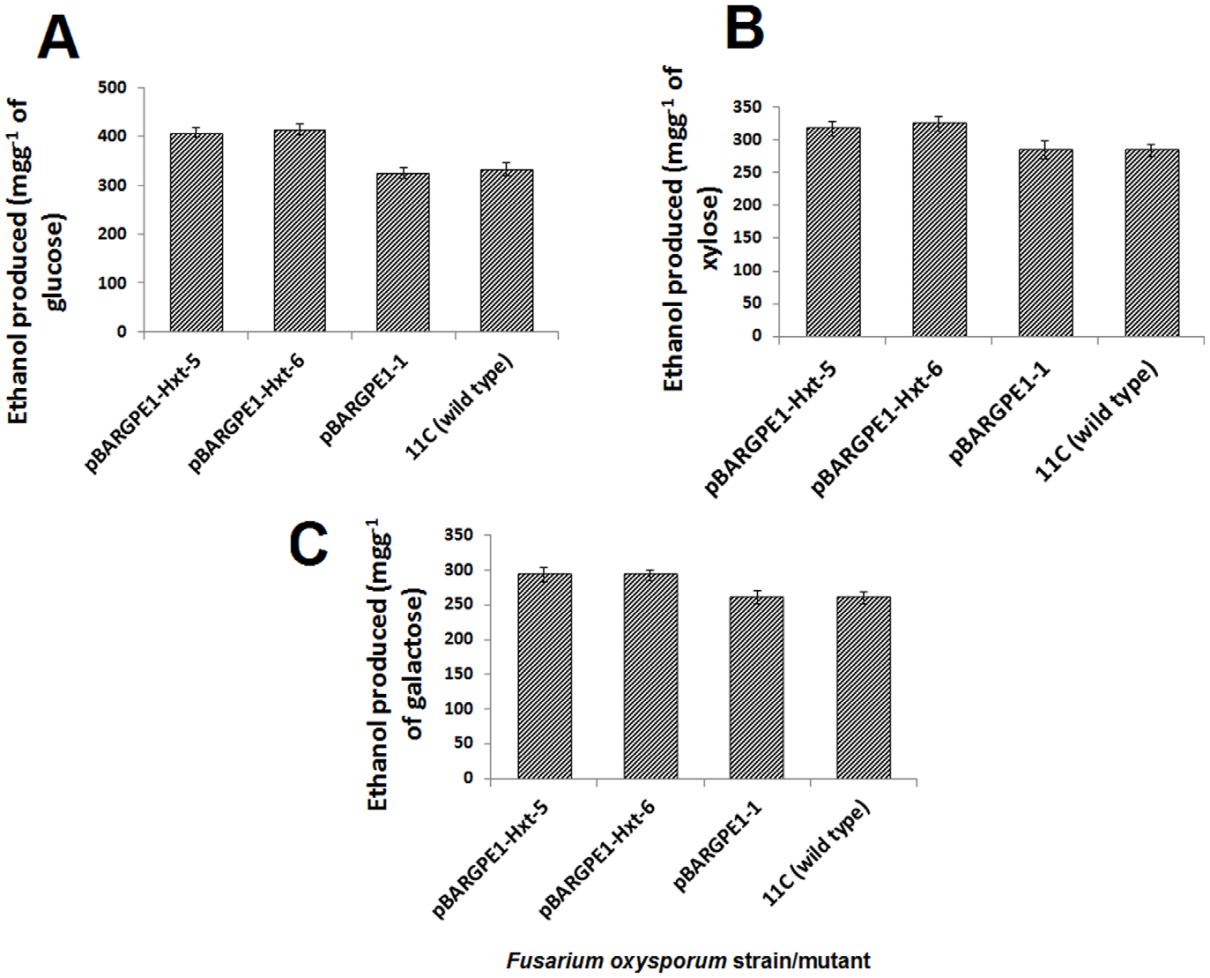
The effect of overexpressing the high affinity glucose transporter (*Hxt*) on the ability of *Fusarium oxysporum* strain 11C to ferment hexose and pentose sugars to ethanol. Wild type fungus, two mutants overexpressing *Hxt* (pBARGPE1-Hxt-5 and pBARGPE1-Hxt-6), and a mutant transformed with the empty silencing vector (pBARGPE1-1) were grown in minimal media shake flask cultures supplemented with (**A**) 10 mM glucose, (**B**) xylose and (**C**) galactose for 24 h aerobic and thereafter 96 h oxygen-limiting growth. Ethanol produced in the culture was estimated using QuantiChrom™ Ethanol Assay Kit (DIET-500) (BioAssay Systems, USA) according to manufacturer’s instruction. Results are based on two experiments, each with three replicates per strain/mutant per medium. Bars indicate SEM (LSD_0.05_ A = 75.12, LSD_0.05_ B = 33.11; LSD_0.05_ C = 31.86).

**Figure 6:**
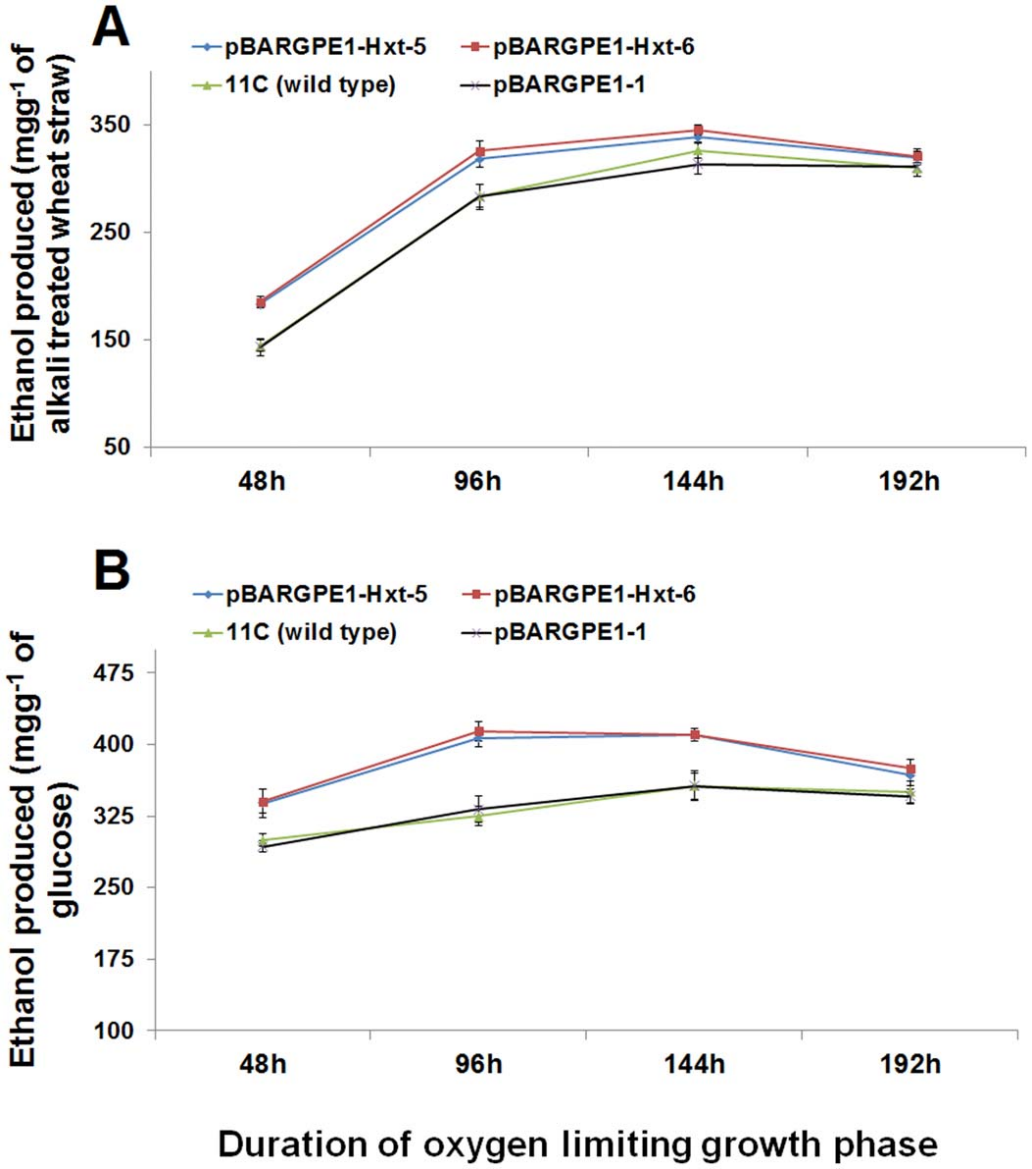
Ethanol production by *Fusarium oxysporum* mutants pBARGPE1-Hxt-5, pBARGPE1-Hxt-6, pBARGPE1-1 and wild type strain 11C during (A) the consolidated bioprocessing (CBP) of alkali treated wheat straw and (B) the fermentation of glucose. For CBP, the shake flask cultivation of delignified straw were conducted as essentially described by Christakopoulos et al. [9] with varying length of the oxygen limiting growth phase. Glucose fermentation was carried out in 100 ml conical flask containing 30 ml minimal media [51] supplemented with 10 mM glucose. For the initial aerobic growth phase, medium was inoculated with fungal spores (10^5^ ml^−1^), plugged with sterile cotton wool and incubated at 30°C in a shaking incubator for 24 h at 150 rpm under dark. Thereafter, flasks were plugged with cork and sealed with parafilm and incubated at 50 rpm, 30°C for different time points. Ethanol produced in both the cultures was estimated using QuantiChrom™ Ethanol Assay Kit (DIET-500) (BioAssay Systems, USA) according to manufacturer’s instruction. Results are based on two experiments, each with three replicates per strain/mutant per medium. Bars indicate the SEM (LSD_0.05_ A = 25.06, LSD_0.05_ B = 29.67).

The two overexpression mutants tested, pBARGPE1-Hxt-5 and pBARGPE1-Hxt-6, produced significantly higher yields of ethanol from glucose (*P*≤0.05), (Figure 5A) xylose (*P*≤0.05) (Figure 5B) and galactose (*P*≤0.05) (Figure 5C) in comparison to either the wild type strain 11C or the mutant transformed with the empty vector (pBARGPE1-1). Overexpression enhanced the rate of ethanol production from alkali-treated straw, with yields reaching a plateau by 96 h incubation under oxygen-limiting conditions (Figure 6A). At this time point, the overexpression mutants, pBARGPE1-Hxt-5 and pBARGPE1-Hxt-6 reached ≥78.55% (≥318.13 mgg^−1^ of alkali-treated straw) of the maximum theoretical yield whereas the wild type and empty vector mutant had yielded ≤70.12% (≤284.00 mgg^−1^ of alkali-treated straw) (Figure 6A). Similarly, overexpression enhanced the rate of ethanol production from glucose (Figure 6B). In the case of the overexpression mutants, pBARGPE1-Hxt-5 and pBARGPE1-Hxt-6, the ethanol yield peaks at 96 h of oxygen-limiting growth, producing ≥80% of the theoretical yield (≥407.14 mgg^−1^ of glucose). But the wild type strain 11C or the mutant (pBARGPE1-1) transformed with the empty vector reached the peak yield (356.5 mgg^−1^ of glucose) after 144 h of oxygen-limited growth (Figure 6B).

### Glucose and Xylose Uptake Kinetics

**Figure 7:**
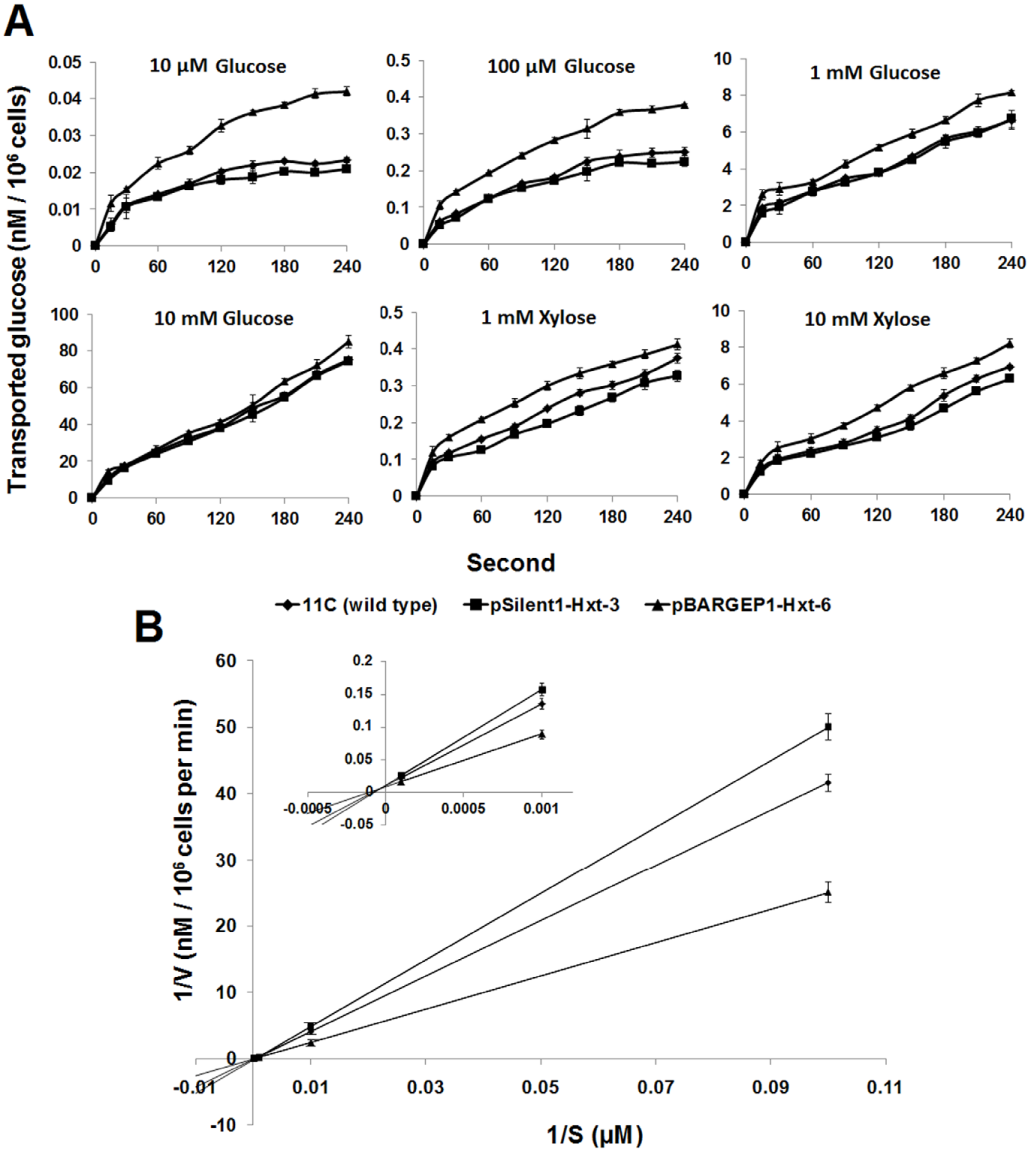
Kinetics of glucose uptake by wild type *Fusarium oxysporum* strain 11C, overexpression mutant pBARGPE1-Hxt-6 and PTGS mutant pSilent1-Hxt-3. (**A**) Uptake of D-glucose and D-xylose (nM of glucose/xylose transported per 10^6^ cells) by *F. oxysporum*. The amount of sugar used in each transport assay is indicated above each graph. Uptake was measured as described by Jørgensen et al. [58]. Results are based on two experiments, each with three replicates per strain/mutant per medium. Bars indicate the SEM. (**B**) Double-reciprocal plots were plotted to deduce the Km_(glucose)_ and Vmax values for the *F. oxysporum* hexose transporter (Hxt). The inset is an amplification of the 1/S values from 0 to 0.001 µM. S, Substrate concentration (µM); V, velocity (nM/10^6^ cells per min). Bars indicate SEM.

The effect of both *Hxt* overexpression and PTGS on both the glucose and xylose transport capacity of *F. oxysporum* was determined. When spores were incubated with 10 and 100 µM glucose concentrations, overexpression mutant pBARGPE1-Hxt-6 was able to transport glucose almost two times faster than the wild-type strain (*P*≤0.01) (Figure 7A). At the higher concentration of 1 mM glucose, the difference between the mutant and wild type decreased (to 1.26 fold more uptake in the former) (*P*≤0.05) (Fig. 7A). Differences between the wild type and PTGS mutant pSilent1-Hxt-3 were very small and only significant at 10 µM glucose concentration (0.9 fold, relative to the wild type; *P*≤0.05). When the data were plotted as double-reciprocal plots (Lineweaver-Burk transformation), differences between the wild type and both overexpression and silencing mutants indicated a Km_(glucose)_ 0.9-0.7 mM for the high-affinity glucose transport component of *F. oxysporum* which is near the range of a high affinity transporter (Km_(glucose)_ 1–2 mM; [18] (Figure 7B). The calculated Vmax (obtained with the double-reciprocal plot) for pBARGPE1-Hxt-6 was 1.85 nM/10^6^ cell/s, whereas the value for the wild-type strain and the silencing mutant were 1.67 nM/10^6^ cell/s and 1.5 nM/10^6^ cell/s respectively.

The Hxt also affected the xylose transport capacity of *F. oxysporum* (Figure 7A). The overexpression mutant transported 1.25-fold more xylose than wild type when incubated under either 1 or 10 mM initial xylose (*P*≤0.05). Conversely, the PTGS mutant pSilent1-Hxt-3 showed significant reduction of at least 14% in xylose transport capacity compare to wild type under both 1 and 10 mM initial xylose concentration (*P*≤0.05) (Figure 7A).

### Effect of Hxt on the Transcription of Other Sugars Transporters during CBP

**Figure 8:**
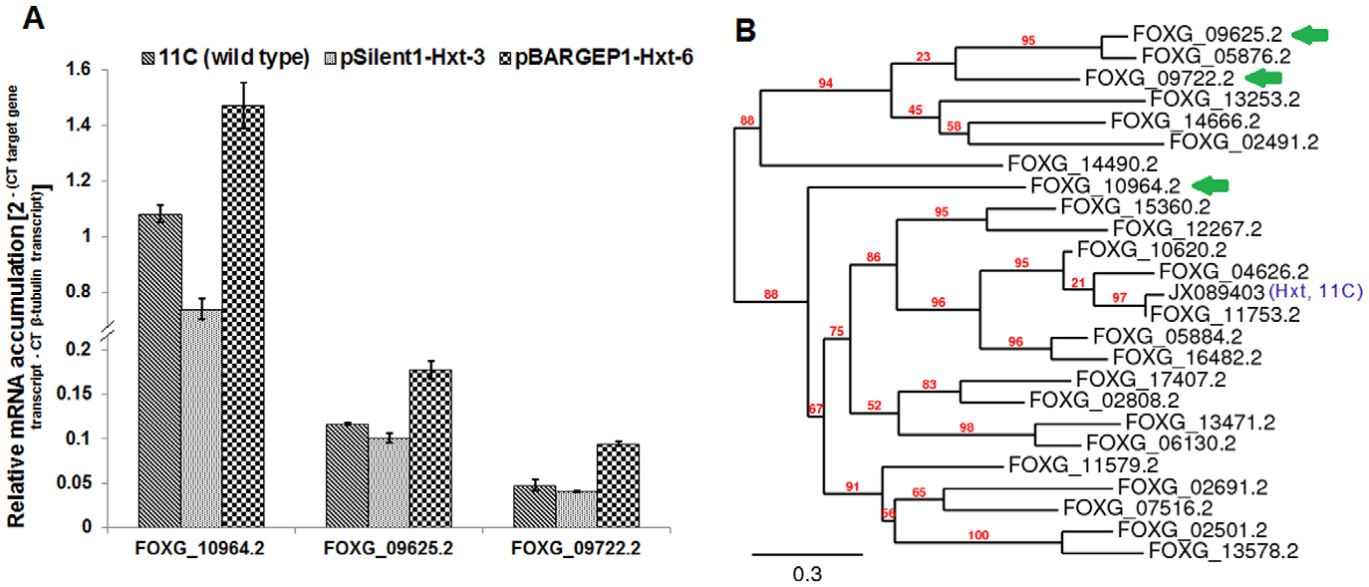
Effect of the Hxt on the transcription of other *Fusarium oxysporum* sugar transporter genes during consolidated bioprocessing (CBP) of wheat straw/bran. Wild type fungus 11C, *Hxt*-silenced mutant pSilent-1-Hxt-3 and overexpression mutant pBARGPE1-Hxt-6 were aerobically cultured on wheat straw/bran (10∶1 ratio) and RT-PCR was conducted using RNA isolated from samples harvested 24 h post-inoculation. (A) Transcript accumulation in RNA extracts was quantified relative to that of the housekeeping gene β-tubulin (FOXG_06228.2) by 2^∧-ΔΔCt^ method, where ΔΔCt = (Ct, Target gene - Ct, β-tubulin). Results are based on two experiments, each with three replicates per strain/mutant per medium. Bars indicate the SEM (LSD_0.05_ = 0.015) (B) A phylogenetic tree was constructed based on the protein sequence of 20 *F. oxysporum* strain 4287 sugar transporters. Protein sequences were aligned using European Bioinformatics Institutes’s ClustalW2 tool (www.ebi.ac.uk) [62] and a distance tree of 100 bootstrapped data sets was generated by using the Phylogeny.fr program and the neighbor-joining method [64]. Arrows indicated the three genes alluded to in part (A) above that were affected by Hxt expression.

The *F. oxysporum* f.sp. *lycopersici* (strain 4287) genome (http://www.broadinstitute.org) encodes twenty four genetically-distinct sugar transporter genes (Table S2). Real time RT-PCR was used to analyse the effect of both overexpression and PTGS of the *Hxt* on the transcription of these genes in *F. oxypsorum* strain 11C during growth on wheat straw/bran. Of the 24, 20 transcripts were detected at 24 h post-fungal inoculation (see Table S2). Transcription of three phyogenetically-distinct genes, a sugar transporter gene (FOXG_10964.2) and two hexose transporters (FOXG_09625.2, FOXG_09722.2), was positively regulated by *Hxt* during the CBP of wheat straw (Figure 8). Under glucose (10 mM for 24 h under the similar condition used for glucose fermentation assay) two of these three genes were not transcribed and the transcription of the other (FOXG_10964.2) was 0.008-fold that of the *Hxt* (results not shown).

## Discussion

This study has shown that a *F. oxysporum Hxt* gene enhances the rate of lignocellulose bioconversion to ethanol. This gene is activated in the initial stages of CBP; this is not surprising as the expression level of functional membrane proteins is usually very low [31] and at transcript level, their detection is only possible within the log phase of growth. The presence of 12 TM helixes in the putative glucose transporter protein identified in the *F. oxysporum* strain 11C indicates that it is a hexose transport (Hxt) protein belonging to the 12-TM transporter superfamily [32], [33].

Like yeast, it was observed that the *F. oxysporum* genome encodes a range of Hxt proteins [17], [34]. In the case of yeast, the sequence identity among the 20 different glucose transporters ranges from 25% to 99.7% and the proteins range in length from 540 to 592 residues; the two exceptions to this are the Rgt2 and Snf3 proteins which are respectively 200 and 300 residues longer at the C-terminal than the other glucose transporters [35]. Snf3 and Rgt2 are the most divergent members of the glucose transporter family, being only ∼25% similar to their relatives [35]. Interestingly, the Hxt protein identified in *F. oxysporum* has more homology with these two proteins than other major yeast Hxt proteins but it lacks their C-terminal extension. This extension distingushes Snf3 and Rgt2 from other transporters as it equips them with the capacity to regulate glucose transport [34], [36]; they act as sensors of extracellular glucose and trigger the generation of an intracellular signal that triggers the induction of other *Hxt* gene expression [34], [36]. Unlike yeast, *F. oxysporum* and other related filamentous fungi do not have any predicted hexose transporters with such long C-terminal extension [34], thus their glucose mediated induction or repression of *Hxt* genes is not regulated by Snf3 or Rgt2 -like proteins. However, its differential expression did affect the transcription of three phylogenetically distinct and uncharacterised sugar transporters during growth on wheat straw/bran. There was no evidence that these sugar transporter genes were regulated by glucose (at least by 10 mM).

Based on the similarity of the transmenbrane domains of the yeast Snf3 and Rgt2 and the *F. oxysporum* Hxt protein we hypothesised that the latter might also be highly sensitive to glucose levels. The mRNA level of the *F. oxysporum Hxt* gene decreased with increasing glucose concentration. As the high affinity glucose transporters are repressed by increasing glucose concentration [17], [20], [21], these results indicate that the encoded *F. oxysporum* Hxt protein is a high affinity glucose transporter. Yeast complementation experiments did not provide conclusive evidence that the fungal gene was a high affinity glucose transporter protein (Ali et al., unpubl. data). A similar problem was faced by other researches when a *Trichoderma harzianum* glucose transporter was expressed in the same null mutant yeast [37]. But glucose uptake studies conducted using fungal overexpression and PTGS mutants provided evidence that the protein under study was a high affinity glucose transporter. The effect of overexpression was greater than that of PTGS; it may be that in the case of PTGS there is compensation by other transporters. In yeast it was observed that deleting even up to seven different glucose transporter genes did not result in any growth defects on glucose [38].

The activity of glucose transporters declines as a result of non-competitive inhibition by the ethanol produced during alcoholic fermentation [39], [40], [41]. By changing the membrane lipid environment, ethanol indirectly inhibits the activity of various transport systems [39], [40]. The rate of hexose transportation is determined by both the activity and the number of hexose transporters in the plasma membrane [28], hence with increasing ethanol concentration *F. oxysporum* cells try to compensate for the reduction in Hxt activity by increasing their numbers. During the bioconversion of straw to ethanol by *F. oxysporum* the amount of glucose in the broth remains very low (unpubl. data) and this is likely to promote the activity of the Hxt protein.

In yeast the decrease in fermentation activity during alcoholic fermentation does not appear to be due to changes in the activities of the glycolytic enzymes [42]. On the contrary, the rate of anaerobic fermentation by resting cells of *S. cerevisiae* is limited by the rate of sugar transport [28], [39]. Thus the enhanced production of Hxt proteins could enhance fermentation capacity. Overexpression of the *Hxt* gene increased the maximum theoretical yield of ethanol from 23.8 to 33.8% in the case of untreated wheat straw/bran. Though this was the highest reported ethanol yield from any unprocessed lignocellulosic material, it is quite below the industrially exploitable yields. But in case of pre-treated straw, overexpression of *Hxt* enhanced the rate of alcohol production and increased the final yield by 5% relative to control empty vector and wild type strains. To our knowledge, this is the highest yield to date by a fungus from pre-treated agricultural waste [3], [4], [6], [7], [9], [43], [44].

Though overexpression of Hxt enhances ethanol production, it was not accompanied by any significant increase in fungal biomass at the end of CBP. It may that fungal biomass production plateaued during the prolonged period of oxygen-limited growth. Under CBP conditions, the concentration of free sugar in the reactor remains low (unpubl. data) and thus the amount of sugar uptake and rate of ethanol formation may reach an equilibrium resulting in minimal sugar conversion to biomass. A previous study showed that there was no correlation between biomass production and CBP efficacy of *F. oxysporum* [8].

Xylose is the second most common sugar found in plants and the inability of current microbial strains to successfully ferment xylose is a significant limiting factor. The primary reason for this inefficient fermentation is that a naturally-occurring yeast or fungal strain equipped with an independent xylose transporter has yet to be identified [45]. The studies carried out herein showed that the *F. oxysporum Hxt* gene directly enhances both glucose and xylose transport. It was further observed that overexpression of the *Hxt* gene enhanced not only glucose, but also xylose and galactose fermentation rates. Transport of D-xylose has been found to be related to D-glucose transport [46], [47] and in yeast it was observed that some of the major glucose transporter like Hxt4, Hxt5, Hxt7 and Gal2 are also involved in xylose transport [48].

In conclusion, the rate of sugar transportation across cell membranes plays a major role in the overall activity and productivity of microorganisms when it comes to producing ethanol from lignocellulosic material. The characterised *F. oxysporum* Hxt is regulated by glucose concentration in the medium and it affects uptake of both C6 and C5 sugars. As overexpression of the *Hxt* gene enhances the CBP activity of *F. oxysporum*, it is clear that like cellulolytic and ethanologenic enzymes, sugar transporters also play a major role in determining the efficiency of microbial bioconversion of lignocellulose to ethanol. Thus overexpression of Hxt proteins should be an integral part of the approach used to develop any highly efficient engineered bioprocessing agents.

## Materials and Methods

### Fungal Strains

*F. oxysporum* strains 11C (IMI501118) and 7E (IMI501116) was used in this study. This strain was isolated from Irish soils as described by Ali et al. [8]. Prior to use, fungi were sub-cultured onto potato dextrose agar (PDA) (Difco, UK) plates and incubated at 25°C for 5 days. Fungal conidial inoculums was produced in mung bean broth as described by Brennan et al. [49] and were resuspended in minimal medium [50] at a concentration of 10^6^ conidia ml^−1^.

### Solid-state Cultivation (SSC) on Unprocessed Straw/Bran

Straw particles (<2 mm diameter) from wheat (*Triticum aestivum* L. cultivar Einstein) were prepared as described by Ali et al. [8]. Straw was blended with 10% (ww^−1^) unprocessed wheat bran (particle size ≤3 mm) (Odlums, Ireland). The SSC involved an initial aerobic phase followed by an oxygen-limiting growth phase and was conducted as described by Ali et al. [8]. For the aerobic growth period, Erlenmeyer flasks were plugged with non-absorbent cotton covered with aluminium foil. For the subsequent oxygen-limited incubation period, flasks were plugged with cork and sealed with parafilm. For RNA isolation to conduct RT-PCR analysis, samples were harvested at 24–96 h post-inoculation under aerobic conditions. For each fungal strain/mutant, three replicate flasks were used and each experiment was conducted twice.

For studies which analysed ethanol production by mutant and wild type fungal strains, mycelia were allowed to grow aerobically on untreated wheat straw/bran blend (9∶1) for 96 h for biomass and saccharolytic enzyme production, followed by 96 h incubation under oxygen-limiting conditions growth to facilitate the fermentation of released sugars into ethanol. Following SSC, ethanol was condensed and collected as described by Ali et al. [8]. For each fungal strain/mutant, three replicate flasks were used and each experiment was conducted twice.

Shake flask studies were used in order to determine the efficacy of *F. oxysporum* mutant strains and wild type strain 11C in producing ethanol from alkali-treated straw. The culture conditions used were those essentially described by Christakopoulos et al. [9].

### Sugar Fermentation

The ability of wild type and mutant strains of *F. oxyporum* to ferment glucose and pentose sugars were determined using shake flask cultures and oxygen-limiting conditions. Flasks (100 ml) contained 30 ml of the minimal media (MM) described by Leung et al. [51], were supplemented with 10–100 mM of sugar. For the initial aerobic growth phase, medium was inoculated with fungal spores (10^5^ ml^−1^), plugged with sterile cotton wool and incubated at 30°C for 24 h at 150 rpm in the dark. Thereafter flasks were plugged with cork and sealed with parafilm and incubated at 50 rpm, 30°C for 24–192 h. The ethanol content of the culture was determined for two subsamples per sample. This experiment was conducted twice and each time it included three replica flasks per fungal strain/mutant.

### RNA Isolation

Mycelial samples were flash-frozen with liquid, freeze-dried and homogenised in a mixer mill (Retsch MM400, Germany) at 30 Hz for 1 min with two 2.3 mm steel beads. RNA was isolated as previously described by Chang et al. [52]. RNA was DNased-treated using TURBO DNA-free kit (Ambion, USA), according to the manufacturers’ recommendations. RNA quality was confirmed by visualising RNA following agarose gel electrophoresis and yields were quantified using a *NanoDrop*® ND-1000 Spectrophotometer, all as described previously [53].

### Real time RT-PCR Analysis

Reverse transcription (RT) of total RNA was conducted as described previously [54], except that the primer used was oligo dT_12–18_ (Invitrogen). The housekeeping gene used for normalisation of real-time RT-PCR data was *β*-tubulin (*β*-tub*; Fusarium* database no FOXG_06228.2); Real-time PCR quantification of target gene and of the housekeeping gene was performed in separate reactions as described previously [12]. See Table S1 for *Hxt*- or *β*-tub-specific primers (Hxt-F1/R1and *β*-tub-F/R respectively) used for real time RT-PCR analysis. Real-time PCR primers specific to other sugar transporter genes were designed using gene sequences from *F. oxysporum* f.sp. *lycopersici* (strain 4287) released by Broad Institute (www.broadinstitute.org) (See Table S1).

### Rapid Amplification of cDNA Ends (RACE)

5′- and 3′-RACE was conducted in order to clone the full-length mRNA sequence of the *Hxt* gene from *F. oxysporum* strain 11C. RACE analysis was conducted using the Clontech SMARTer™ RACE kit (Clontech Laboratories Inc., USA), according to the manufacturers’ protocols and gene-specific primers RACE-Hxt-MF/MR (see Table S1). RACE products were gel-purified using the same SMARTer™ RACE kit, cloned using the pGEM-T Easy “TA” cloning kit (Promega, USA) according to the manufacturers’ protocol and sequenced (Macrogen, Korea). The ORF was determined using NCBI ORF finder (http://www.ncbi.nlm.nih.gov/projects/gorf/) and was used to query the deduced amino acid sequence (http://web.expasy.org/translate) against protein sequences in the *Saccharomyces* Genome Databas*e* (SGD) (http://www.yeastgenome.org/) and the *Fusarium* comparative genomics database (FCGD) (http://www.broadinstitute.org/annotation/genome/fusarium).

### Construction of the RNA Silencing Vector

Post-transcriptional gene silencing (PTGS) was used to generate mutants of *F. oxysporum* strain 11C silenced in the *Hxt* gene function. The silencing vector pSilent-1-Hxt (See Figure S6) was constructed using the pSilent-1 vector [55], which contains Aspergillus nidulans *trp*C promoter and terminator flanking two MCS that are separated by an intron from a *Magnaporthe grisea* cutinase gene and hyg as a selectable marker gene which provides resistance to hygromycin. A 415 bp fragment of the *Hxt* gene with appropriate overhanging restriction sites was inserted into each of the two MCS in the sense (upstream of the intron) or antisense (downstream of the intron) direction (See Figure S6). Primers Si_Hxt-L-F2/R2 and Si_Hxt-R-F2/R2 were respectively used to amplify the sense and antisense direction inserts (see Table S1). The PCR amplification reactions were carried out as mentioned by Ali et al. [12]. Products and plasmid were digested (see Table S1 for enzymes) (New England Biolabs, USA), ligated (T4 DNA ligase, Promega, USA) and the correct alignment of sense and antisense segments were confirmed by partial sequencing of the plasmid using ACpSi-F/R primers (see Table S1), designed to anneal to the end of *trpC* promoter and the beginning of *trpC* terminator, respectively.

### Construction of the Over Expression Vector

The *Hxt* overexpression vector pBARGPE1-Hxt (See Figure S7) was constructed using the pBARGPE1 vector [56] which contains *A. nidulans gpdA* promoter and *trpC* terminator flanking a MCS and bar as a selectable marker gene which provides resistance to Basta (active ingredient = phosphinothricin). Primers FL_Hxt-F/R were used to amplify the 1596 bp ORF plus the 77 bp 3′-UTR sequence of the *Hxt* gene with appropriate overhanging restriction sites (see Table S1). The PCR amplification reaction (50 µl) contained 5 µl 5′-RACE ready cDNA (of *F. oxysporum* strain 11C), 5 µl 10×LA PCR Buffer (Mg_2_+ plus), 0.5 µl (2.5 unit) TaKaRa LA Taq DNA polymerase (Takara, Japan), 8 µl (2.5 mM each) dNTPs mix and 0.2 µM each of the forward and reverse primers. Amplifications were performed in a Peltier Thermal Cycler (PTC) –200 DNA Engine (MJ Research) with the following conditions: initial denaturation for 60 s at 94°C was followed by 38 cycles of 98°C for 10 s, 60°C for 30 s, 68°C for 105 s with a final extension of 10 min at 72°C. Products and plasmid were digested (see Table S1 for enzymes) (New England Biolabs, USA), ligated (T4 DNA ligase, Promega, USA) and the correct alignment of the gene was confirmed by partial sequencing of the plasmid using ACpBg-F/ACpSi−/R primers (see Table S1), designed to anneal to the end of *gpdA* promoter and the beginning of the *trpC* terminator, respectively.

### Generation of Fungal Mutants

*F. oxysporum* strain 11C was transformed with pSilent-1-Hxt and pBARGPE1-Hxt in order to respectively silence and over express the *Hxt* gene. Transformations were also performed with empty vectors pSilent-1 and pBARGPE1 to generate negative control mutants. Protoplasts were produced from fungal spores as previously described [12]. Protoplasts were transformed with the appropriate vector as described by Doohan et al. [57]. Following selection on PDA (Oxoid, UK) or minimal medium, [51] containing 60 µgml^−1^ hygromycin (Sigma, Germany) [57] for silencing or 1000 µgml^−1^ phosphinothricin (Sigma, Germany) [51], for overexpression respectively. Putative transformants were subcultured five times on the same selective medium, then four times on non-selective medium and finally, transformant stability was verified by growing on the selective PDA/minimal medium. Fungal mycelium generated from a single spore was subcultured on PDA and transferred to a 15% vv^−1^ glycerol solution for storage at −70°C. Transformation was confirmed by both PCR and southern blot analysis (See Materials and Methods S1 for PCR and southern blot analysis).

### Glucose Uptake Assay

MM [51] supplemented with10 mM glucose as a carbon source was inoculated with conidia of *F*. *oxysporum* to a final concentration of 10^6^ spores ml^−1^. Cultures were incubated for 15 h at 30°C. Swollen spores were collected by centrifugation at 4°C and washed four times with MM without a carbon source. Finally, spores were resuspended in twofold concentrated in MM. To begin each uptake assay, 100 ml pre warmed spore culture broth was transferred to 100 ml pre warmed MM containing either glucose (20 µM, 200 µM, 2 mM or 20 mM) or xylose (2 or 20 mM) in the shake flask reactor. The assay was performed at 30°C and lasted 5–6 min. Samples were taken every 15–30 s, using the device described by Jørgensen et al. [58]. Determination of specific glucose and xylose uptake and estimation of uptake parameters were as described by Jørgensen et al. [58]. Experiments were performed twice, each time including three replicates per treatment.

### Estimation of Ethanol Yield and Biomass Produced from Straw/Bran

Ethanol (mgg^−1^ substrate) was determined using the QuantiChrom™ Ethanol Assay Kit (DIET-500) (BioAssay Systems, USA) according to manufacturer’s instructions. Fungal biomass levels were determined based on the chitin-derived glucosamine content of the solid culture component. Chitin was hydrolysed into *N*-acetyl glucosamine as previously described [59], which was then assayed by the modified colorimetric method described by Ride & Drysdale [60].

### Statistical Analysis

See Materials and Methods S1 for information regarding data distribution, transformation and pooling. The significance of treatment effects was analysed within the Statistical Package for the Social Sciences (SPSS 11.0, SPSS Inc.) by either (i) normally distributed data - one-way ANOVA with Post Hoc pair wise Least Significance Difference (LSD) comparisons (P = 0.05), or (ii) non-normally-distributed data - the Kruskal-Wallis H test [61]. Correlations between mean values from different normally-distributed data sets were calculated using Pearson product moment analysis.

## Supporting Information

Figure S1 **Crystal structure and transmembrane domains of the** ***F. oxysporum*** **Hxt.**
(DOCX)

Figure S2 **Sequence similarity among the** ***F. oxysporum*** **Hxt and the Rgt2 and Snf3 proteins of yeast.**
(DOCX)

Figure S3 **Confirmation of uptake and genomic integration of** ***Hxt*** **gene silencing plasmid into** ***F. oxysporum*** **.**
(DOCX)

Figure S4 **Confirmation of uptake and genomic integration of** ***Hxt*** **gene overexpression plasmid into** ***F. oxysporum.***
(DOCX)

Figure S5 **Analysis of the accumulation of transcript encoding Hxt in wild type and gene-silenced/overexpressing mutants.**
(DOCX)

Figure S6 **The silencing vector pSilent-1-Hxt.**
(DOCX)

Figure S7 **The over expression vector pBARGPE1-Hxt.**
(DOCX)

Materials and Methods S1 **Selection of fungal mutants and statistical analysis.**
(DOCX)

Results S1 **Gene silenced and overexpression mutants.**
(DOCX)

Table S1 **Primers used in the study.**
(DOCX)

Table S2 **Effete of Hxt expression on the transcription of other sugar transporter genes of** ***F. oxysporum.***
(DOCX)
