# Supplementary material for: Insights from the Fungus Fusarium oxysporum Point to High Affinity Glucose Transporters as Targets for Enhancing Ethanol Production from Lignocellulose
Source: PLoS One. 2013 Jan 30;8(1):e54701. doi: 10.1371/journal.pone.0054701 (PMC3559794; doi:10.1371/journal.pone.0054701)
Supplement: Figure S1 — Crystal structure and transmembrane domains of the F. oxysporum Hxt. (DOCX) [file pone.0054701.s001.docx]

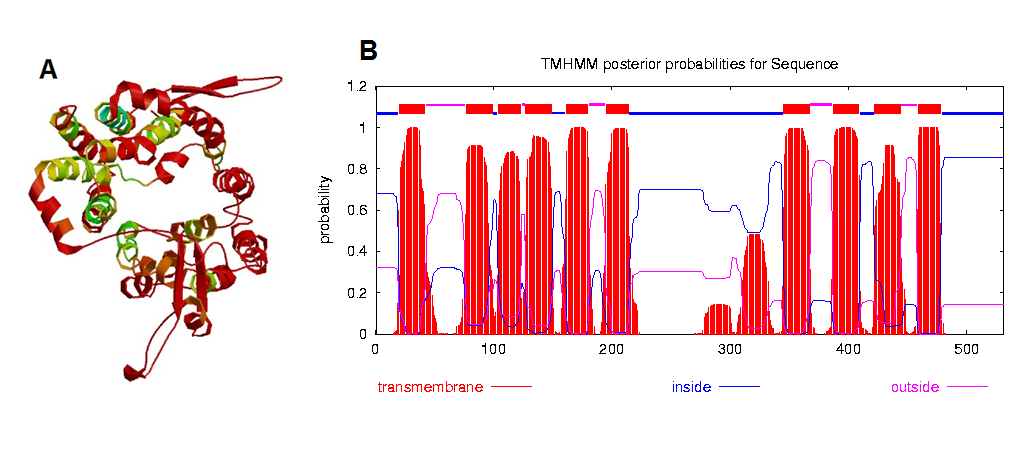


**Figure S1.** **Crystal structure and transmembrane domains (TMD) of the *Fusarium oxysporum* hexose transporter.** **(A)** The putative crystal structure was based on a template of lactose permease (2cfqA) [1] (*E*value 1.3E-26). Colour indicates residue error; QMEAN Z-Score: -10.175 [2]. The putative crystal structure was generated using SWISS-MODEL Workspace (http://swissmodel.expasy.org) [3]. **(B)** The TMDs were predicted with the TMHMM Server version 2.0 (http://www.cbs.dtu.dk/services/TMHMM/).

**Reference:**

1. Kaback HR, Iwata S, Mirza O, Verner G, Guan L (2006) Structural evidence for induced fit and a mechanism for sugar/H+ symport in LacY. EMBO J: 1177-1183.

1. Benkert P, Biasini M, Schwede T (2011) Toward the estimation of the absolute quality of individual protein structure models. Bioinformatics 27: 343-350.

3. Arnold K, Bordoli L, Kopp J, Schwede T (2006) The SWISS-MODEL workspace: a web-based environment for protein structure homology modelling. Bioinformatics 22: 195-201.
