# Supplementary material for: Insights from the Fungus Fusarium oxysporum Point to High Affinity Glucose Transporters as Targets for Enhancing Ethanol Production from Lignocellulose
Source: PLoS One. 2013 Jan 30;8(1):e54701. doi: 10.1371/journal.pone.0054701 (PMC3559794; doi:10.1371/journal.pone.0054701)
Supplement: Figure S2 — Sequence similarity among the F. oxysporum Hxt and the Rgt2 and Snf3 proteins of yeast. (DOCX) [file pone.0054701.s002.docx]

**Figure S2.** Sequence similarity among the glucose transporter proteins (Hxt) identified in *Fusarium oxysporum* strain 11C and the Rgt2 and Snf3 transporter proteins of Saccharomyces cerevisiae (yeast). Deduced amino acid sequence of the *F. oxysporum* *hxt* gene was generated by the Expasy Translate tool (http://web.expasy.org/translate/). Yeast glucose transporter amino acid sequences were obtained from [Saccharomyces Genome Database](http://www.yeastgenome.org/) (www.yeastgenome.org)*.* Protein sequences were aligned using European Bioinformatics Institutes’s ClustalW2 tool (www.ebi.ac.uk) [1].

**Reference**

1. Larkin M, Blackshields G, Brown N, Chenna R, McGettigan P, et al. (2007) Clustal W and Clustal X version 2.0. Bioinformatics 23: 2947.
