# Supplementary material for: Insights from the Fungus Fusarium oxysporum Point to High Affinity Glucose Transporters as Targets for Enhancing Ethanol Production from Lignocellulose
Source: PLoS One. 2013 Jan 30;8(1):e54701. doi: 10.1371/journal.pone.0054701 (PMC3559794; doi:10.1371/journal.pone.0054701)
Supplement: Figure S3 — Confirmation of uptake and genomic integration of Hxt gene silencing plasmid into F. oxysporum . (DOCX) [file pone.0054701.s003.docx]

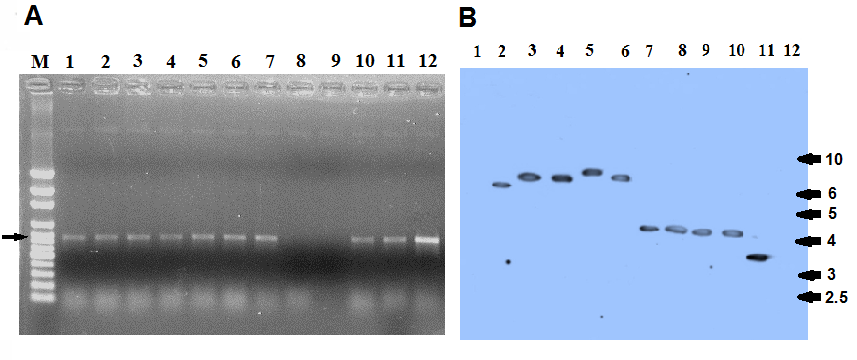


**Figure S3. Confirmation of uptake and genomic integration of *Hxt* gene silencing plasmid pSilent-1-Hxt into *Fusarium oxysporum.*** (**A**) PCR was used determine if fungal mutant genomic DNA extracts contained the hygromycin (*hyg*) gene present in the pSilent1 backbone. Lanes represent: 1 to 7, gDNA from mutants pSilent-1-Hxt-1 to 7 transformed with the *Hxt-*silencing vector; 8, gDNA from wild type fungus strain 11C; 9, no DNA (negative control); 10-11, gDNA from mutant pSilent-1-*A* & *B* transformed with the empty silencing vector; 12, plasmid pSilent1 DNA (positive control). Arrow indicates *hyg* PCR product (747bp). **(B)** Southern blot analysis was used to confirm plasmid integration in *Sac*I- & *Kpn*I-digested fungal genomic DNA and to determine gene copy number, using a 747nt fragment of the *hyg* gene as a probe. Lanes 1 – 6 represent DNA digested with *sac*I and 7-12 DNA digested with both *Sac*I and *Kpn*I; 1& 12, gDNA from wild type fungus strain 11C; 2 & 11, gDNA from mutant pSilent-1-*A* transformed with the empty silencing vector; 3 – 6 and 7-10, gDNA from mutants pSilent-1-Hxt-1, pSilent-1-Hxt-3, pSilent-1-Hxt-5 and pSilent-1-Hxt-6. Arrows indicate molecular size (Kb) based on the 1kb DNA ladder (Solis BioDyne, Estonia).
