# Supplementary material for: Insights from the Fungus Fusarium oxysporum Point to High Affinity Glucose Transporters as Targets for Enhancing Ethanol Production from Lignocellulose
Source: PLoS One. 2013 Jan 30;8(1):e54701. doi: 10.1371/journal.pone.0054701 (PMC3559794; doi:10.1371/journal.pone.0054701)
Supplement: Figure S4 — Confirmation of uptake and genomic integration of Hxt gene overexpression plasmid into F. oxysporum. (DOCX) [file pone.0054701.s004.docx]

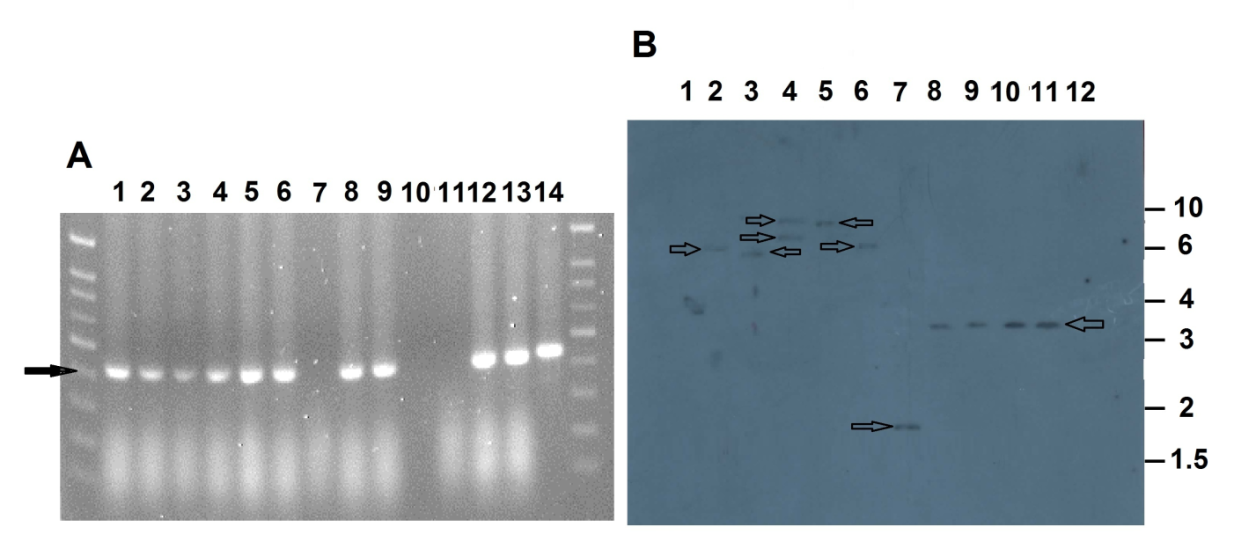


**Figure S4. Confirmation of uptake and genomic integration of *Hxt* gene overexpression plasmid pBARGPE1-Hxt into *Fusarium oxysporum.*** (**A**) PCR was used determine if fungal mutant genomic DNA extracts contained the *bar* gene present in the pBARGPE1 backbone. Lanes represent: 1 to 9, gDNA from mutants pBARGPE1-Hxt-1 to 9 transformed with the *Hxt-*over expression vector; 10 no DNA (negative control); 11, gDNA from wild type fungus strain 11C; 12-13, gDNA from mutant pBARGPE1-1 & 2 transformed with the empty over expression vector; 14, plasmid pBARGPE1 DNA (positive control). Arrow indicates *bar* PCR product (433bp). **(B)** Southern blot analysis was used to confirm plasmid integration in *Sma*I and *Pml*I -digested fungal genomic DNA and to determine gene copy number, using a 433nt fragment of the *bar* gene as a probe. Lanes 1 – 6 represent DNA digested with *Sma*I and 7-12 DNA digested with both *Sma*I and *Pml*I; 1 & 12, gDNA from wild type fungus strain 11C; 2-5 & 8-11, gDNA from mutants pBARGPE1-Hxt-1, pBARGPE1-Hxt-5, pBARGPE1-Hxt-6 and pBARGPE1-Hxt-9; 6 & 7, gDNA from mutant pBARGPE1-1 transformed with the empty over expression vector (digested with SmaI); Arrows indicate molecular size (Kb) based on the 1kb DNA ladder (Solis BioDyne, Estonia).
