# Supplementary material for: Insights from the Fungus Fusarium oxysporum Point to High Affinity Glucose Transporters as Targets for Enhancing Ethanol Production from Lignocellulose
Source: PLoS One. 2013 Jan 30;8(1):e54701. doi: 10.1371/journal.pone.0054701 (PMC3559794; doi:10.1371/journal.pone.0054701)
Supplement: Figure S5 — Analysis of the accumulation of transcript encoding Hxt in wild type and gene-silenced/overexpressing mutants. (DOCX) [file pone.0054701.s005.docx]

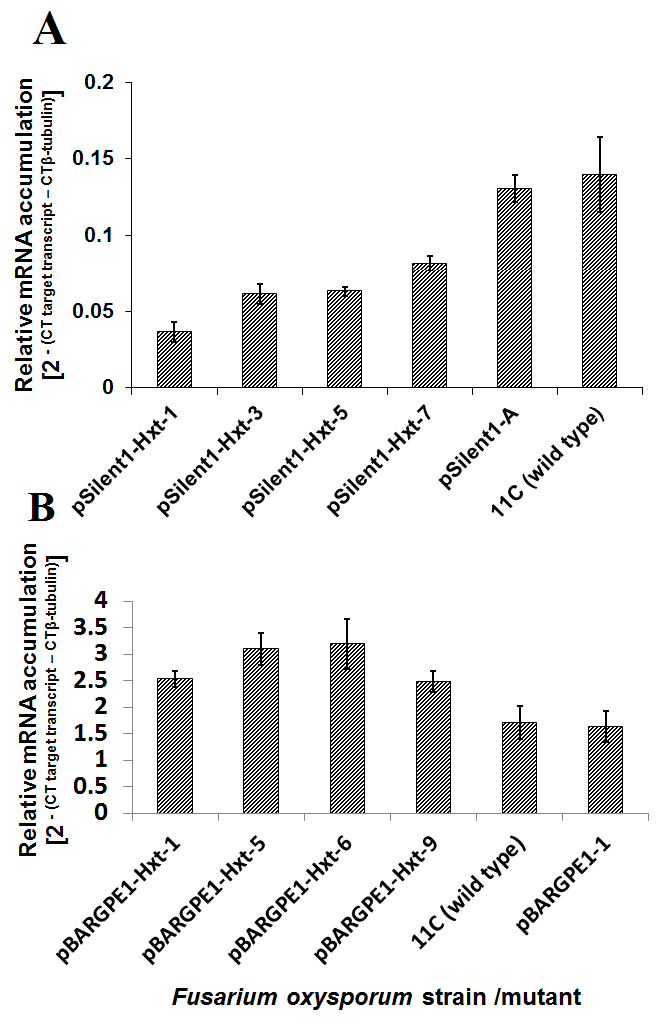


**Figure S5. Analysis of the accumulation of transcript encoding a high affinity glucose transporter (Hxt) in wild type and gene-silenced/overexpressing mutants of *Fusarium oxysporum* strain 11C.** **(A)** Gene silenced mutants pSilent-1-Hxt-1, 3, 5 & 7, mutant pSilent-1-*A* transformed with the empty silencing vector and wild type fungus; (**B)** gene overexpressed mutants pBARGPE1-Hxt-1, 5, 6 & 9, mutant pBARGPE1-1 transformed with the empty over expression vector and wild type fungus. For both A and B, fungi were aerobically cultured on a straw/bran (10:1 ratio) mix for 24h. *Hxt* transcript accumulation in RNA extracts was quantified relative to that of the housekeeping gene β-tubulin (FOXG_06228.2). Results are based on two experiments, each with three replicates per treatment. Bars indicate standard error of mean (LSD_0.05_ A = 0.0247, LSD_0.05_ B = 0.662).
