# Supplementary material for: Insights from the Fungus Fusarium oxysporum Point to High Affinity Glucose Transporters as Targets for Enhancing Ethanol Production from Lignocellulose
Source: PLoS One. 2013 Jan 30;8(1):e54701. doi: 10.1371/journal.pone.0054701 (PMC3559794; doi:10.1371/journal.pone.0054701)
Supplement: Figure S6 — The silencing vector pSilent-1-Hxt. (DOCX) [file pone.0054701.s006.docx]

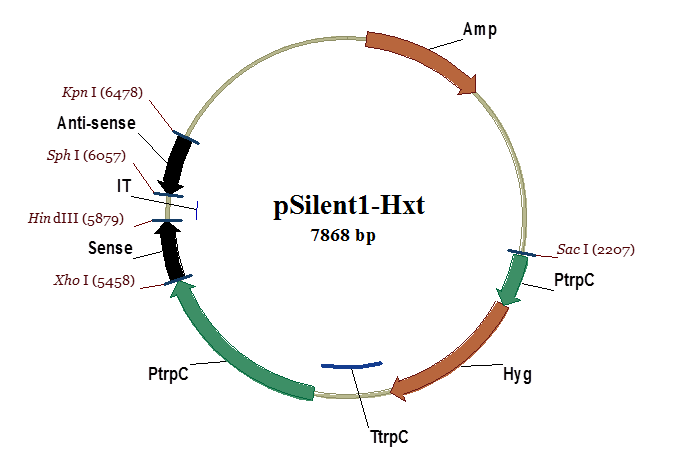


**Figure S6.** The silencing vector pSilent-1-Hxt was constructed using the pSilent-1 vector [1] which contains *Aspergillus nidulans* *trp*C promoter and terminator flanking two MCS separated by an intron from a *Magnaporthe grisea* cutinase gene. A 415bp fragment of the *Hxt* gene with appropriate overhanging restriction sites (as shown in the figure) was inserted into each of the two MCS in the sense (upstream of the intron) or antisense (downstream of the intron) direction. Vector diagram was generated using Vector NTI Advance® 11.5 software (Invitrogen).

**Reference**

1. Nakayashiki H, Hanada S, Quoc NB, Kadotani N, Tosa Y, et al. (2005) RNA silencing as a tool for exploring gene function in ascomycete fungi. Fungal Genetics and Biology 42: 275-283.
