# Supplementary material for: Insights from the Fungus Fusarium oxysporum Point to High Affinity Glucose Transporters as Targets for Enhancing Ethanol Production from Lignocellulose
Source: PLoS One. 2013 Jan 30;8(1):e54701. doi: 10.1371/journal.pone.0054701 (PMC3559794; doi:10.1371/journal.pone.0054701)
Supplement: Figure S7 — The over expression vector pBARGPE1-Hxt. (DOCX) [file pone.0054701.s007.docx]

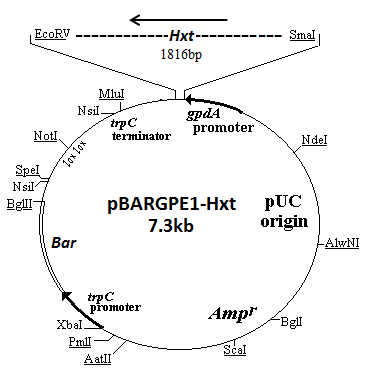


**Figure S7.** The over expression vector pBARGPE1-Hxt was constructed using the pBARGPE1 vector [1] which contains Aspergillus nidulans *gpdA* promoter and *trpC* terminator flanking a MCS and bar as a selectable marker gene which provides resistance to basta or phosphinothricin. The 1672bp ORF sequence of the *hxt* gene with appropriate overhanging restriction sites was inserted into the MCS (as shown in the figure).

**Reference**

1. Pall M, Brunelli J (1993) A series of six compact fungal transformation vectors containing polylinkers with multiple unique restriction sites. Fungal Genet Newsl 40: 59-62.
