# Supplementary material for: Insights from the Fungus Fusarium oxysporum Point to High Affinity Glucose Transporters as Targets for Enhancing Ethanol Production from Lignocellulose
Source: PLoS One. 2013 Jan 30;8(1):e54701. doi: 10.1371/journal.pone.0054701 (PMC3559794; doi:10.1371/journal.pone.0054701)
Supplement: Materials and Methods S1 — Selection of fungal mutants and statistical analysis. (DOCX) [file pone.0054701.s008.docx]

**Material and Methods S1**

Selection of fungal mutants

The PCR target/Southern blot probe was a fragment of the *hyg* gene for silencing mutants and of the *bar* gene for overexpression mutants. DNA was isolated from wild type/mutants as previously described [1]. For putative silencing mutants, PCR was conducted using *hyg-*specific primers (Hyg-F1/R1; see supplementary Table S1) which were used to amplify a 747bp *hyg* gene fragment from 100ng gDNA from putative mutants or 50ng pSilent-1 plasmid DNA (as a positive control and to produce probe for southern blot analysis). For the putative overexpression mutants PCR was conducted using *bar-*specific primers, (Bar-F1/R1; see supplementary Table S1), which were used to amplify a 433bp *bar* gene fragment from 100ng gDNA of putative mutants or 50ng pBARGPE1 plasmid DNA (as a positive control and to produce probe for southern blot analysis). The PCR reaction components and the conditions were as described already in the manuscript. PCR products (10μl) were electrophoresed through 1% (wt vol^-1^ TAE) agarose gels containing 0.5 μg ml^−1^ ethidium bromide and visualised using Imagemaster VDS and Liscap software (Pharmacia Biotech, USA).

For southern blot analysis of mutants, genomic DNA (10µg) from wild type *F. oxysporum* strain 11C and putative mutants was digested overnight with restriction enzymes (New England Biolabs, USA). Blots of putative gene-silenced mutants were prepared using DNA digested with either *Sac*I (single digest) or *Sac*I plus *Kpn*I (double digest). Blots of putative overexpression mutants were prepared using DNA digested with either *Sma*I (single digest) or *Sma*I plus *Pml*I (double digest). Digests were electrophoresed through 0.8% (wt vol^-1^ TAE) agarose gels (at 30volt; overnight) and blotted onto reinforced NC membrane (Optitran BA-S85, Schleich & Schuell, Germany) as described by Sambrook et al. [2]. A747bp fragment of the *hyg* gene and a 433bp fragment of the *bar* gene (amplified by PCR as described above) was respectively used as probes for Southern blot analysis of putative gene silenced and overexpression mutants. The AlkPhos Direct Labeling and Detection System with CDP-Star (GE Healthcare, USA) were used for labelling and detecting the 747bp *hyg* probe. The hybridisation and detection procedures were performed according to the manufacturer’s instructions.

Statistical analysis

All RT-PCR data, glucose and xylose uptake data and ethanol yield data from glucose and alkali-treated wheat straw experiments were non-normally distributed, as determined using the Ryan Joiner test [3] within Minitab (Minitab release 13.32^©^, 2000 Minitab Inc.). All other data sets were normally distributed. All data except the temporal analysis of *Hxt* transcript accumulation, glucose and xylose uptake data could be transformed to fit a normal distribution using the Johnson transformation [3] within Minitab (Minitab release 13.32^©^, 2000 Minitab Inc.). The homogeneity of data sets across replicate experiments was confirmed by two-tailed correlation analysis (non-normal data: Spearman Rank; normal data: Pearson product moment) conducted within the Statistical Package for the Social Sciences (SPSS 11.0, SPSS Inc.) (*r* ≥ 0.721; *P =* 0.01) [4]. Therefore, data sets from the replicate experiments were pooled for the purposes of further statistical analysis.

**Reference**

1. Ali SS, Khan M, Mullins E, Doohan F (2012) Identification of Fusarium oxysporum genes associated with lignocellulose bioconversion competency. Manuscript under submission.

2. Sambrook J, Fritsch EF, Maniatis T (2001) Molecular cloning: Cold Spring Harbor Laboratory Press Cold Spring Harbor, NY:.

3. Ryan T, Joiner BL (1983) Normal probability plots and tests for normality. Minitab Statistical Software: Technical Reports. The Pennsylvania State University, State College, PA. Available from MINITAB, Inc.

4. Snedecor G, Cochran W (1980) Statistical Methods, 6th edn (Ames, Iowa State University Press).
