# Supplementary material for: Insights from the Fungus Fusarium oxysporum Point to High Affinity Glucose Transporters as Targets for Enhancing Ethanol Production from Lignocellulose
Source: PLoS One. 2013 Jan 30;8(1):e54701. doi: 10.1371/journal.pone.0054701 (PMC3559794; doi:10.1371/journal.pone.0054701)
Supplement: Results S1 — Gene silenced and overexpression mutants. (DOCX) [file pone.0054701.s009.docx]

**Results S1**

Gene silenced and overexpression mutants

PTGS and gene overexpression via fungal transformation was used to respectively repress and up-regulate the function of the Hxt in *F. oxysporum* strain 11C. Transformation with empty vector pSilent-1 or with pSilent-1-Hxt was confirmed by PCR analysis of the *hyg* gene (Supplementary Figure S3A) and by Southern analysis using a PCR-amplified segment of the *hyg* gene as a probe (Supplementary Figure S3B). Southern hybridisation confirmed that four silencing mutants (pSilent-1-Hxt-1, 3, 5 and 6) contained a single copy of the vector integrated into the genomic DNA. Transformation with empty overexpression vector pBARGPE1 or with pBARGPE1-Hxt was confirmed by PCR analysis of the *bar* gene (Supplementary Figure S4A) and Southern blot analysis of five mutants using a PCR-amplified segment of the *bar* gene as a probe confirmed that all the mutants except pBARGPE1-Hxt-5 has a single copy of the vector integrated into the genomic DNA (Supplementary Figure S4B). The mutant pBARGPE1-Hxt-5 genome carried two copies of the plasmid. The efficacy of gene silencing and overexpression was verified by RT-PCR analysis of the *hxt* transcript levels in fungi cultured for 24h on straw/bran under aerobic conditions. There was almost a 2 to 4-fold decrease in the expression level of *Hxt* in the silencing mutants as compared to either the wild type strain 11C or the mutant (pSilent-1-A) transformed with the empty vector (Supplementary Figure S5A). In the case of the overexpression mutants, there was almost a 2-fold increase in the *Hxt* transcript levels in mutants pBARGPE1-Hxt-5 and pBARGPE1-Hxt-6 as compared to the wild type strain 11C or the mutant (pBARGPE1-1) transformed with the empty vector (Supplementary Figure S5B).
