# Supplementary material for: Insights from the Fungus Fusarium oxysporum Point to High Affinity Glucose Transporters as Targets for Enhancing Ethanol Production from Lignocellulose
Source: PLoS One. 2013 Jan 30;8(1):e54701. doi: 10.1371/journal.pone.0054701 (PMC3559794; doi:10.1371/journal.pone.0054701)
Supplement: Table S1 — Primers used in the study. (DOCX) [file pone.0054701.s010.docx]

**Table S1.** Primers used in the study.

| **Primer ID** | Target gene | **Forward primer (5΄ - 3΄)** | **Reverse primer (5΄ - 3΄)** |
| --- | --- | --- | --- |
| Hxt-F1/R1 | *Hxt* | TGCATTGCATTTGTTTGGTT | CTGTCGATCCTCCCAAGAGTT |
| RACE-Hxt-MF/MR | *Hxt* | TCGACTTGGGTCTTCAACCT | CGCCGAGGTTCTTGATAAAA |
| *β*-tub-F/R | β-tubulin | CAACAACATCCAAACAGCCC | CTCACCAACACGCTTGAAGA |
| Hyg-F1/R1 | Hygromycin | TTCCGGAAGTGCTTGACATT | TTCTACACAGCCATCGGTCC |
| ACpSi-F/R | *trp*C promoter/terminator | ACGACCCGGTCATACCTTCT | AAACAGCTTGACGAATCTGGA |
| Si_Hxt-L-F2/R2 | *Hxt* | CTCTCGAGGGTTTGGCTGCTGTTTTCTC | AAAAGCTCATCCCGCCAACTCTTCATC |
| Si_Hxt-R-F2/R2 | *Hxt* | CTGGTACCGGTTTGGCTGCTGTTTTCTC | AAGCATGCATCCCGCCAACTCTTCATC |
| FL_Hxt-F/R | *Hxt* | ACCCGGGATGGGACTTATCGGCAAAGTG | AGATATCACAACCATTGCCTCCAAAAG |
| ACpBg-F | *gpdA* promoter | TCAGTTCGAGCTTTCCCACT |  |
| Bar-F1/R1 | *Bar* | GCACCATATCGTCAACCACTACATCG | AGCTGCCAGAAACCACGTCATG |
| FLpC210_Hxt-F/R | *Hxt* | GAACATATGGGACTTATCGGCAAAGTG | CGAGCATGCACAACCATTGCCTCCAAAAG |
| ACpC210-F/R | *SSA2/ CYCP1* promoter/terminator | CAATTGGGCTGGGTTTTCT | GTACGGGCGTGTGGTCTAGT |
| FOXG_11753.2 _F1/R1 | High affinity glucose transporter | GCTACTCAACTGGGCTCTGG | ACCCAGTGAACCGACTTACG |
| FOXG_10620.2_F1/R1 | High affinity glucose transporter | CGTGCCAAGTCCCTCAGTAT | GAAGTAGACGAAGGCGATGC |
| FOXG_04626.2_F1/R1 | MFS Monosaccharide transporter | TCGCTTGAGGAAGTTGACCT | TTGCTCTCATGGTGTGAAGC |
| FOXG_17407.2_F1/R1 | Monosaccharide transporter | TTTTCTTCTTCTGGGGAGCA | AATTACGCCACCCTTCTCCT |
| FOXG_15360.2_F1/R1 | Monosaccharide transporter | GCGCCTTCGTCTACACCTAC | TCTTAGACGTGGTCGTCGTG |
| FOXG_05884.2_F1/R1 | RCO3 | GGAGATGTCGAGGATCAGGA | CCCTTACTCTCGGTGCGTAG |
| FOXG_16482.2_F1/R1 | RCO3 | TGTTGCCCATCACATCAACT | GCCGTCACTTCACTCCTCTC |
| FOXG_06130.2_F1/R1 | High affinity glucose transporter | GGAGCAGATTGACGAGATG | CGATCCGTGTGCATTATCTG |
| FOXG_10964.2_F1/R1 | Sugar transporter | CTGCCGTCAGAAGTCTTTCC | GCACCAGGATCCGTAAAAGA |
| FOXG_09722.2_F1/R1 | Hexose transporter | CGAATTGGGCTTTCAACACT | CCAAGCTGGAGTACCGATGT |
| FOXG_02491.2_F1/R1 | Hexose transporter | CGCCTTTATCCATCGCTTAC | GTTTCAGGGAACAGGAACCA |
| FOXG_05876.2_F1/R1 | Hexose transporter | AAGGGACCTCGTGCTACTGA | GCGCATCATGAAGTGAATTG |
| FOXG_14666.2_F1/R1 | MFS hexose transporter | TCCTCGCAGTTCTTTTCGTT | CGCCCTTCTGCTCAGTATTC |
| FOXG_13253.2_F1/R1 | MFS hexose transporter | CAACCAGACCAACCCTATCG | CGTTAGCATCATCACCATCG |
| FOXG_07516.2_F1/R1 | Hexose carrier protein | ACTTGGGCATTCGTACTGCT | CATCCTTCCCCTCAAACAGA |
| FOXG_02691.2_F1/R1 | Hexose carrier protein | TCCCATCATGATCTCCAACA | CGAGACCAAGTCGAATAGCC |
| FOXG_13578.2_F1/R1 | Hexose carrier protein | AGCGATAGGAATCAGCTCCA | CCTCCAGCTTGAAGTTGCTC |
| FOXG_13471.2_F1/R1 | Hexose carrier protein | ATCGAAAACATTGGCTACCG | TCAAAACCACCTCGAAGACC |
| FOXG_02501.2_F1/R1 | Hexose transporter | TCTTCGGGTCTTGGTGTTTC | CCTTCTTCAGCCCATACTCG |
| FOXG_11579.2_F1/R1 | Hexose carrier protein | TACATCGTGTACGCCGTCAT | TGATCAGCCTTTTCCTTGCT |
| FOXG_09625.2_F1/R1 | Hexose transporter | TCTCCGTCATGTACGTCAGC | TCAAAGATGGCCTGGATTTC |
| FOXG_12267.2_F1/R1 | High affinity glucose transporter | CGTCAACCCCATCACTTCTT | CGTCCGTTCCAATCGTTACT |
| FOXG_14490.2_F1/R1 | MFS Monosaccharide transporter | ATCACTCCCGTGTCCATCTC | ACTCCCCAGTTTCACCACTG |
| Primers were design using the Primer3 software (version 0.4.0; http://frodo.wi.mit.edu/primer3/). | | | |
