# Supplementary material for: Insights from the Fungus Fusarium oxysporum Point to High Affinity Glucose Transporters as Targets for Enhancing Ethanol Production from Lignocellulose
Source: PLoS One. 2013 Jan 30;8(1):e54701. doi: 10.1371/journal.pone.0054701 (PMC3559794; doi:10.1371/journal.pone.0054701)
Supplement: Table S2 — Effete of Hxt expression on the transcription of other sugar transporter genes of F. oxysporum. (DOCX) [file pone.0054701.s011.docx]

| **Table S2.** Effete of Hxt expression on the transcription of other sugar transporter genes of *Fusarium oxysporum* | | | | |
| --- | --- | --- | --- | --- |
| Sager transporter gens^1^ | *Fusarium* database no. | Relative mRNA accumulation after 24h aerobic growth on a straw/bran (10:1 ratio) mix.^2^ | | |
|  |  | 11C (wild type) | pSilent1-Hxt-3 | pBARGEP1-Hxt-6 |
| Regulator of conidiation genes-3 (RCO3) | FOXG_05884.2 | 2.252 ± 0.119 | 2.356 ± 0.195 | 2.3 ± 0.129 |
| Sugar transporter | FOXG_10964.2 | 2.187 ± 0.067 | *1.524 ± 0.076 | *2.946 ± 0.163 |
| High affinity glucose transporter | FOXG_11753.2 | 0.486 ± 0.014 | 0.495 ± 0.042 | 0.486 ± 0.032 |
| High affinity glucose transporter | FOXG_10620.2 | 0.285 ± 0.025 | 0.295 ± 0.012 | 0.289 ± 0.009 |
| Monosaccharide transporter | FOXG_02808.2 | 0.175 ± 0.005 | 0.174 ± 0.013 | 0.180 ± 0.011 |
| MFS hexose transporter | FOXG_14666.2 | 0.158 ± 0.009 | 0.157 ± 0.014 | 0.158 ± 0.01 |
| Regulator of conidiation genes-3 (RCO3) | FOXG_16482.2 | 0.141 ± 0.007 | 0.142 ± 0.02 | 0.149 ± 0.014 |
| Hexose transporter | FOXG_09625.2 | 0.116 ± 0.001 | *0.101 ± 0.009 | *0.177 ± 0.005 |
| High affinity glucose transporter | FOXG_12267.2 | 0.074 ± 0.005 | 0.061 ± 0.004 | 0.082 ± 0.005 |
| MFS hexose transporter | FOXG_13253.2 | 0.071 ± 0.005 | 0.071 ± 0.005 | 0.075 ± 0.003 |
| Monosaccharide transporter | FOXG_15360.2 | 0.044 ± 0.002 | 0.044 ± 0.002 | 0.049 ± 0.002 |
| Hexose transporter | FOXG_09722.2 | 0.048 ± 0.006 | *0.041 ± 0.000 | *0.095 ± 0.003 |
| Monosaccharide transporter | FOXG_17407.2 | 0.0021 ± 0.0003 | 0.0022 ± 0.0002 | *0.003 ± 0.0002 |
| Hexose carrier protein | FOXG_11579.2 | 0.013 ± 0.001 | 0.015 ± 0.001 | 0.016 ± 0.001 |
| Hexose transporter | FOXG_02491.2 | 0.006 ± 0.000 | 0.005 ± 0.001 | 0.006 ± 0.001 |
| Hexose transporter | FOXG_02501.2 | 0.0037 ± 0.0004 | 0.0033 ± 0.0005 | 0.0034 ± 0.0003 |
| Hexose carrier protein | FOXG_13471.2 | 0.0017 ± 0.0002 | 0.0017 ± 0.0003 | *0.0029 ± 0.0002 |
| Hexose carrier protein | FOXG_07516.2 | 0.0017 ± 0.0002 | 0.0017 ± 0.0001 | 0.0021 ± 0.0003 |
| Hexose transporter | FOXG_05876.2 | 0.0005 ± 0.0002 | 0.0005 ± 0.0001 | *0.0016 ± 0.0006 |
| MFS Monosaccharide transporter | FOXG_04626.2 | 0.0001 ± 0.0000 | 0.0000 ± 0.0000 | 0.0001 ± 0.0000 |
| MFS Monosaccharide transporter | FOXG_14490.2 | No expression | No expression | No expression |
| High affinity glucose transporter | FOXG_06130.2 | No expression | No expression | No expression |
| Hexose carrier protein | FOXG_02691.2 | No expression | No expression | No expression |
| Hexose carrier protein | FOXG_13578.2 | No expression | No expression | No expression |
| ^1^Genes were selected from the *F. oxysporum*f. sp. *lycopersici*(strain 4287) genome which was sequenced and annotated by the Broad Institute (http://www.broadinstitute.org/annotation/genome/fusarium_group/MultiHome.html).  ^2^Transcript accumulation in RNA extracts was quantified relative to that of the housekeeping gene β-tubulin (FOXG_06228.2) by 2^^-∆∆Ct^ method, where ∆∆Ct=(Ct, Target gene - Ct, β-tubulin). ± indicate standard error of mean, an ‘*’ indicate that values are significantly different from wild type 11C at *P* ≤ 0.05. | | | | |
